# Rock inhibition promotes Na_V_1.5 sodium channel-dependent SW620 colon cancer cell invasiveness

**DOI:** 10.1038/s41598-020-70378-3

**Published:** 2020-08-07

**Authors:** Lucile Poisson, Osbaldo Lopez-Charcas, Stéphanie Chadet, Emeline Bon, Roxane Lemoine, Lucie Brisson, Mehdi Ouaissi, Christophe Baron, Pierre Besson, Sébastien Roger, Driffa Moussata

**Affiliations:** 1 EA4245 Transplantation, Immunologie, Inflammation, Université de Tours, 10 Boulevard Tonnellé, 37032 Tours, France; 2 Inserm UMR1069, Nutrition, Croissance et Cancer, Université de Tours, Tours, France; 3 CHRU de Tours, Tours, France; 4 Institut Universitaire de France, Paris, France

**Keywords:** Cancer, Membrane biophysics

## Abstract

The acquisition of invasive capacities by carcinoma cells, i.e. their ability to migrate through and to remodel extracellular matrices, is a determinant process leading to their dissemination and to the development of metastases. these cancer cell properties have often been associated with an increased Rho-ROCK signalling, and ROCK inhibitors have been proposed for anticancer therapies. In this study we used the selective ROCK inhibitor, Y-27632, to address the participation of the Rho-ROCK signalling pathway in the invasive properties of SW620 human colon cancer cells. Contrarily to initial assumptions, Y-27632 induced the acquisition of a pro-migratory cell phenotype and increased cancer cell invasiveness in both 3- and 2-dimensions assays. This effect was also obtained using the other ROCK inhibitor Fasudil as well as with knocking down the expression of ROCK-1 or ROCK-2, but was prevented by the inhibition of Na_V_1.5 voltage-gated sodium channel activity. Indeed, ROCK inhibition enhanced the activity of the pro-invasive Na_V_1.5 channel through a pathway that was independent of gene expression regulation. In conclusions, our evidence identifies voltage-gated sodium channels as new targets of the ROCK signalling pathway, as well as responsible for possible deleterious effects of the use of ROCK inhibitors in the treatment of cancers.

## Introduction

Colorectal cancer is the third most commonly diagnosed cancer in human, and the incidence of this cancer is constantly progressing in the population, thus representing a serious global health problem^1^. One main cause of patient mortality from colorectal cancer is the development of metastases in distant organs such as in liver, in lungs or in the peritoneum, following the dissemination of cancer cells from the primary tumour^2^. This represents a late and a potential incurable stage of the disease. Although the metastatic progression is a non-linear phenomenon that encompasses dormancy and selection phases, and is a consequence of complex cellular interplays between cancer cells and non-cancer cells of the tumour and of the different organs concerned, it is primarily permitted by the acquisition by cancer cells of pro-invasive capacities. These capacities rely on cancer cell abilities to dissociate from each other (subsequently to the loss of cell–cell contacts), to migrate through and/or digest components of the extracellular matrix (ECM), to penetrate surrounding tissues, to reach and to survive into the lymphatic or blood circulation, before seeding in a secondary organ^3^. Therefore, in metastatic progression, the increased motility of cancer cells and their interaction with the extracellular matrix are critical events that are directly dependent on the controlled remodelling of the cytoskeleton^4^. Rho GTPases are a family of small GTPases belonging to the Ras superfamily that, when bound to GTP, recruit a range of different effectors playing important roles in the dynamic regulation of focal adhesion, acto-myosin contraction and cell motility^5,6^. Among them, RhoA has been identified to play a central role in regulating the actin organization, in acto-myosin contraction, in cancer cell adhesion and migration. This is mainly achieved through the activation of the RhoA main effectors Rho-associated coiled-coil kinases (ROCK) which are serine/threonine protein kinases consisting of two isoforms ROCK-1 and ROCK-2^7,8^. Indeed, ROCK phosphorylate different substrate proteins such as LIM Kinases^9^, ezrin/radixin/moesin^10^, myosin phosphatase^11^ or myosin light chain (MLC)^12^, which promote the formation of actin stress fibres, associate the cytoskeleton to the plasma membrane and generate contractile forces^13^.

The expression of ROCK has been shown to be increased in several cancers, to correlate with a bad prognosis, and their activity has been demonstrated to substantially contribute to cancer progression^14–16^. Furthermore, several somatic mutations in genes encoding ROCK-1 or ROCK-2, leading to a gain-of-function, have been identified in several cancers and especially in colorectal cancer^17,18^. As such, ROCK are generally perceived as key players in cancer development and progression^8,19^, which led to consider the use of ROCK inhibitors in the treatment of cancers^7,20,21^. Indeed, the use of ROCK inhibitors such as Y-27632 or Fasudil (HA-1077) decreased the migration and invasion capacities of several cancer cell types^22,23^, among which colon cancer cells^19,24^. However, several other studies reported a pro-cancerous effect of ROCK inhibition by promoting the growth and migration of some cancer cells^25,26^, such as a gain in colon cancer cell proliferation and invasion^27–29^. Signalling pathways involved in such deleterious effects are still elusive, and certainly need to be better understood.

In colorectal cancer, voltage-gated sodium channels (Na_V_), and notably the Na_V_1.5 isoform, have been demonstrated to be abnormally upregulated and to participate to carcinoma cell invasiveness^30–32^. The functional link between Na_V_ and ROCK signalling in colorectal cancer has not been investigated so far.

In this study, we explored the effect of ROCK inhibition, on the growth and invasiveness of SW620 human colon cancer cells, in both 2- and 3-dimensions models. We show that both the pharmacological and molecular inhibition of ROCK-1 and ROCK-2 induce SW620 cancer cell invasiveness, by promoting the activity of the pro-invasive voltage-gated sodium channel Na_V_1.5 activity.

## Results

### ROCK inhibitor Y-27632 promotes the acquisition of a migratory phenotype and invasive capacities in SW620 human colon cancer cells

**Figure 1:**
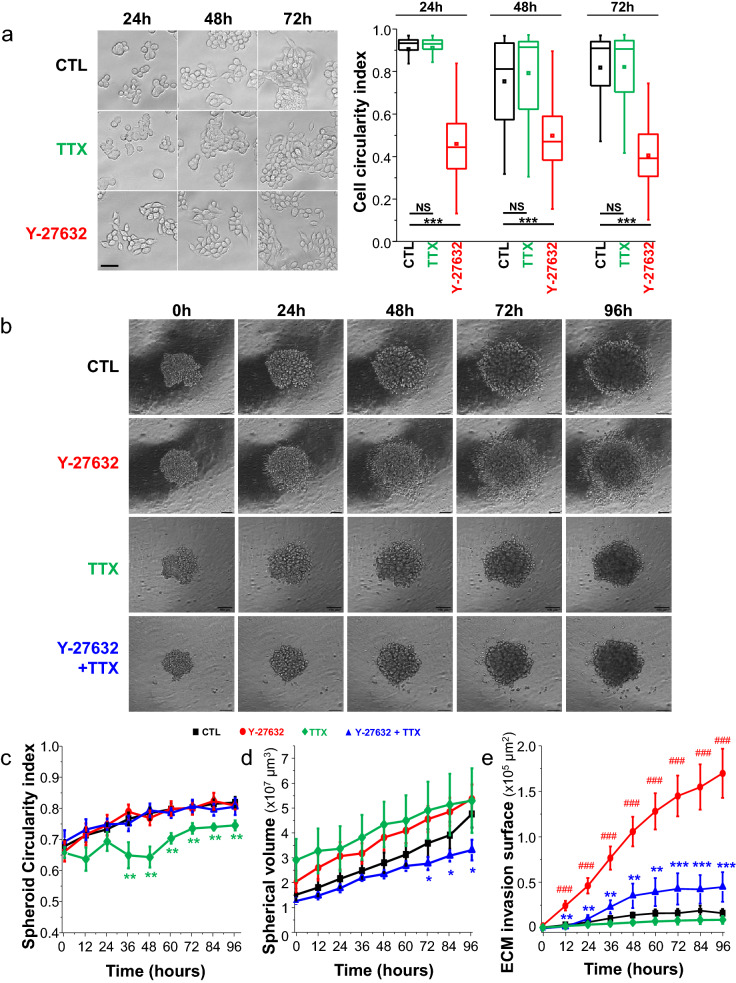
The ROCK inhibitor Y-27632 enhances 3D colon cancer cell invasiveness. (**A**) Left, representative micrographs of SW620 human colon cancer cells in control condition (vehicle 0.1% DMSO, CTL) or treated for 24 h, 48 h or 72 h in presence of 30 µM TTX or 10 µM Y-27632. Scale bar, 30 µm. Right, a cell circularity index was calculated from micrographs taken in absence or presence of Y-27632 at the three different times (n = 100 cells for each condition). This was performed using the Fiji software after having manually delineated the shape of cells. ***Statistical difference at *P* < 0.001 (Mann–Whitney rank sum test) versus CTL condition of corresponding time. NS stands for not statistically different. (**B**) Representative phase contrast micrographs (× 10 amplification objective) taken from SW620 colon cancer cells grown as spheroids in a 3-dimension matrix composed of Matrigel™. Pictures of spheroids were taken at different incubation times (0 h, 24 h, 48 h, 72 h, 96 h ) in control condition (CTL, vehicle) or treated with Y-27632 (10 µM), NaV channel inhibitor tetrodotoxin (TTX, 30 µM), or both (Y-27632 + TTX). Scale bar, 100 µm. (**C**) A spheroid circularity index was calculated over time from time-lapse micrographs in the four experimental conditions indicated above, CTL (vehicle, black line), Y-27632 (10 µM, red line), TTX (30 µM, green line) and the combination Y-27632 + TTX (30 µM, blue line) (n = 12, 8 and 5 spheroids per condition, respectively). Only the TTX group showed as significant reduction of circularity compared to the CTL condition, starting at time 36 h (**P < 0.01, Mann–Whitney rank sum test). There was no other statistical difference between groups. (**D**) Spherical volumes were calculated over time from time-lapse micrographs in the three experimental conditions indicated in B, CTL (vehicle), Y-27632 (10 µM), TTX (30 µM) and Y-27632 + TTX (30 µM) (n = 12, 8 and 5 spheroids per condition, respectively). The co-treatment with Y-27632 and TTX significantly reduced the volume of spheroids as compared to the treatment with Y-27632 alone, or with TTX alone, at times indicated on the figure (*P < 0.05, Mann–Whitney rank sum test). There was no other statistical difference. (**E**) The surface of extracellular matrix (ECM) invasion by SW620 cancer cells, at distance from spheroids, was calculated over time in the four experimental conditions indicated in C. The treatment with Y-27632 significantly increased the invasion area as compared to the CTL condition (^#^*P* < 0.001, Mann–Whitney rank sum test). The co-treatment with Y-27632 and TTX significantly reduced the surface of invasion as compared with the treatment with Y-27632 alone (**P < 0.01; ***P*P*< 0.001, Mann–Whitney rank sum test), but did not differ from the CTL condition.

To characterize the participation of the ROCK-dependent signalling pathway in the invasive capacity of human colon cancer cells, SW620 cells were treated with the potent ROCK-1 and ROCK-2 inhibitor Y-27632 at a concentration of 10 µM. This concentration is conventionally used in both in vitro and in vivo experiments to inhibit ROCK activity^33,34^. We first assessed the effect of such a treatment over time on colon cancer cell morphology, and identified that it was responsible for the acquisition of a more elongated shape, characterized by a significant reduction of the cell circularity index (Fig. 1A). While there might be some differences according to the cell type and mode of migration^35^, an elongated morphology of cancer cells is generally associated with a mesenchymal invasive phenotype^36^. Voltage-gated sodium channels (Na_V_) have been demonstrated to be critical inductors of carcinoma cell invasiveness^37,38^. In colon^30–32^ as in breast cancer^36,39–43^, the Na_V_1.5 isoform has been identified to be abnormally upregulated and associated with invasive and metastatic potencies, and to control cell morphology. Its inhibition leads to an increased circularity in breast cancer cells^36,40^, while its expression and activity promotes the acquisition of an elongated mesenchymal phenotype^43^. Therefore we also tested the effect of the Na_V_ inhibitor tetrodotoxin (TTX) used at a concentration of 30 µM that blocks > 90% Na_V_1.5 currents (Suppl. Figure 1), but identified no significant effect in the morphology of these cells harbouring a roundish morphology (Fig. 1A). Then, we compared the capacity of cancer cells to invade extracellular matrices in control conditions or in presence of Y-27632. For this purpose, SW620 spheroids were grown in a Matrigel-composed 3-dimensional matrix (Fig. 1B). Morphology and growth of spheroids were analysed over a total time duration of 96 h by time-lapse microscopy, along with the capacity of cells to disseminate from spheroids and to invade the matrix. We also tested the effect of TTX alone, and of the combination of both Y-27632 and TTX. Y-27632 induced no significant change in spheroid morphology, assessed by calculating a circularity index (Fig. 1C), or growth (Fig. 1D) as compared to the control condition. However, cancer cells treated with Y-27632 demonstrated a significant 3-time increase in the capacity to disseminate from the spheroid and to invade the extracellular matrix (ECM) (Fig. 1E). Importantly, the use of the Na_V_ inhibitor tetrodotoxin (TTX) used at a concentration of 30 µM prevented the induction of ECM invasion mediated by Y-27632 (Fig. 1E), while having no or mild effect on spheroid circularity and growth (Fig. 1C, D). At the concentrations used, neither Y-27632 nor TTX interfered with SW620 cell viability (Suppl. Figure 2A). These results showed that the ROCK inhibitor Y-27632 unexpectedly induces 3D invasion of SW620, at least partially in dependence on Na_V_ activity.

**Figure 2:**
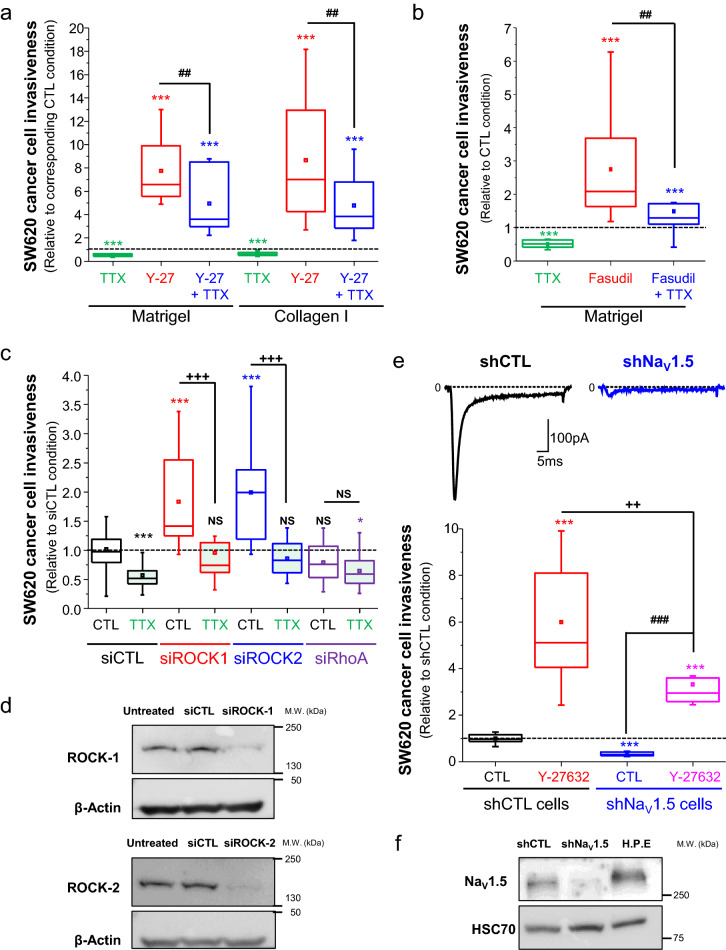
ROCK inhibitors enhance 2D colon cancer cell invasiveness dependently on Na_V_1.5 channels. (**A**) Summary of SW620 colon cancer cell invasiveness studies performed either on Matrigel-coated (300 µg/mL) or Collagen I-coated (500 µg/mL) invasion inserts in control condition (vehicle, 0.1% DMSO), in presence of TTX (30 µM), Y-27632 (10 µM) or the combination Y-27632 + TTX. Results, from 7–8 independent experiments, are expressed relative to the CTL condition which appears as a dashed line. ***P < 0.001 compared to CTL condition (Mann–Whitney rank sum test); ^##^*P* < 0.01 compared to the condition performed in presence of TTX (Mann–Whitney rank sum test). (**B**) Summary of SW620 colon cancer cell invasiveness studies performed on Matrigel-coated (300 µg/mL) invasion inserts in control condition, in presence of TTX (30 µM), Fasudil (20 µM) or the combination Fasudil + TTX. Results, from 8 independent experiments, are expressed relative to the CTL condition (vehicle, 0.1% DMSO) which appears as a dashed line. ***P < 0.001 compared to CTL condition (Mann–Whitney rank sum test); ^##^*P* < 0.01 compared to the condition in presence of TTX (Mann–Whitney rank sum test). (**C**) Effect of silencing the expression of ROCK-1, or ROCK-2 or RhoA using specific siRNA (siROCK1, siROCK2 or siRhoA, respectively), compared to the transfection of irrelevant siRNA (siCTL), on SW620 colon cancer cell invasiveness. These experiments were performed in absence (CTL) or presence of TTX (30 µM). Results are expressed as ratios of mean results obtained with siCTL cells in CTL condition (vehicle). The dashed line indicates a ratio of 1. Results are from 12 to 15 independent experiments and were analysed using Mann–Whitney rank sum tests. ***P < 0.001 compared to CTL condition in siCTL cells; *P < 0.05 compared to CTL condition in siCTL cells; ^+++^*P* < 0.001 when comparing TTX to the CTL condition in siROCK1 cells or in siROCK2 cells. NS stands for not statistically different. (**D**) Representative Western blotting analysis of ROCK-1 (upper panel) or ROCK-2 (lower panel) protein expression in untreated SW620 cells, in cells transfected with irrelevant siRNA (siCTL), or with siROCK1 (upper panel), or with siROCK2 (lower panel) for 48 h. β-actin was used as loading control protein. These blots are representative of four independent experiments. (**E**) Upper panel, representative fast inward sodium currents recorded in shCTL SW620 cells (left) in response to a membrane depolarizing step from − 100 to − 5 mV for 50 ms. This current was absent in SW620 cancer cells stably expressing a short hairpin RNA targeting S*CN5A* gene expression (shNa_V_1.5 cells, right). Lower panel, effect of Y-27632 (10 µM) on cell invasiveness of SW620-sh*CTL* and SW620-shNa_V_1.5 cancer cells. Results are expressed as ratios of mean results obtained with shCTL cells in CTL condition (vehicle). The dashed line indicates a ratio of 1. Results are from 9 independent experiments and were analysed using Mann–Whitney rank sum tests. ***P < 0.001 compared to CTL condition in shCTL cells; ^++^*P* < 0.01 compared to Y-27632 condition in shCTL cells, ^###^*P* < 0.001 compared to the CTL condition in shNa_V_1.5 cells. (**F**) Representative western blotting analysis of Na_V_1.5 protein expression in shCTL and shNa_V_1.5 cell lines, as compared to rat Heart Protein Extract (H.P.E) used as a positive control. HSC70 was used as loading control protein. This blot is representative of three independent experiments.

We then performed 2D-invasion assays using transwell inserts containing an 8-µm-pore-sized filter that we covered by either Matrigel (300 µg/mL) or collagen I (500 µg/mL). These contain characteristic components of ECM that are generally invaded by epithelial cancer cells during the metastatic process, Matrigel mimicking the basement membrane while collagen I mimics ECM of conjunctive tissues. In both cases, the inhibition of Na_V_ activity using TTX reduced cancer cell invasiveness by about 40–50% (median factor of 0.51 on Matrigel, and of 0.58 on collagen I) compared to the control condition, while Y-27632 induced a significant increase of cancer cell invasiveness (by a median factor of 6.6 on Matrigel, and of 7.0 on collagen I). Again, the co-administration of TTX partially prevented (by approximately 45%) the effect of Y-27632 (Fig. 2A). To decipher whether this effect could be due to non-specific effect of Y-27632, or rather due to the inhibition of ROCK, we tested the effect of another selective and potent ROCK inhibitor, Fasudil, on the 2D-invasion of Matrigel-coated inserts. Fasudil (20 µM) also induced an increase of SW620 invasiveness by a median factor of 2.1, albeit responsible for a reduction of cell viability (Suppl. Figure 1B). The effect of Fasudil on cell invasiveness was prevented by approximately 40% in presence of TTX (Fig. 2B). Then, we reduced the expression of either ROCK-1 or ROCK-2, using a mix of three specific sequences of silencing RNA for each target (siROCK-1 or siROCK-2) and compared to the transfection of a null-target siRNA (siCTL). These experiments resulted in a decrease of ROCK-1 or ROCK-2 mRNA expression, as assessed by RT-qPCR, by 67.3 ± 2.3% (n = 3) and by 50.1 ± 2.3 (n = 3), respectively. These reduced expressions mediated by siRNA were confirmed at the protein level by western blotting experiments (Fig. 2D) and we recorded a reduced protein expression compared to the siCTL condition of 70.7 ± 14.8% (n = 4) and 54.5 ± 18.4% (n = 4) for ROCK-1 and ROCK-2, respectively. In siCTL cells, TTX reduced the invasive capacity by about 50% (median 0.52), indicating the participation of Na_V_ in basal invasiveness (Fig. 2C). Knocking down the expression of ROCK-1 increased the invasive capacity of SW620 cancer cells by a median factor of 1.42, and this effect was prevented by TTX. Knocking down the expression of ROCK-2 also increased the invasive capacity of SW620 cancer cells, by a median factor of 1.99. Again, this effect was prevented by the use of TTX. By contrast to these results, knocking down the expression of RhoA, by 75.7 ± 0.3% (n = 3) as assessed by qPCR, using a mix of three specific sequences of silencing RNA (siRhoA), had no effect on SW620 cancer cell invasiveness (Fig. 2C). Taken together, these results suggested that the promotion of SW620 cancer cell invasiveness was dependent on Na_V_ activity and indeed due to the inhibition of both ROCK-1 and ROCK-2. Interestingly, both Y-27632 and Fasudil reduced MDA-MB-231 human breast cancer cell circularity (Suppl. Figure 2C), and Y-27632 increased the invasive capacity of these cells, in which Na_V_1.5 has been demonstrated to promote pro-metastatic capacities^40,44^, by a median factor of 1.51 (Suppl. Figure 1D).

To address the specific regulation of the Na_V_1.5 channel, encoded by the *SCN5A* gene, which has been previously identified as an important enhancer of SW620 cancer cell invasiveness^30,31^, we developed two cell lines derived from SW620, one stably expressing a small hairpin RNA specific for targeting *SCN5A* gene expression (shNa_V_1.5) and the other stably expressing a null-target small hairpin RNA (shCTL). As shown in Fig. 2E (top panel), a fast inward sodium current could be recorded in shCTL but not in shNa_V_.1.5 cells. These two cell lines were treated with Y-27632 (10 µM) or its vehicle (CTL) and cancer cell invasiveness through Matrigel-coated inserts was assessed. As anticipated, in CTL condition, shNa_V_1.5 cells demonstrated a 65%-lower invasion capacity compared to shCTL cells. Furthermore, the Y-27632-mediated induction of invasion was 2.5-fold lower in shNa_V_1.5 cells compared to shCTL cells (Fig. 2E, lower panel). The reduced expression level of Na_V_1.5 proteins in shNa_V_1.5 cells was also confirmed by western blotting (Fig. 2F).

### ROCK inhibitors increase Na_V_1.5 protein expression and activity in SW620 human colon cancer cells

**Figure 3:**
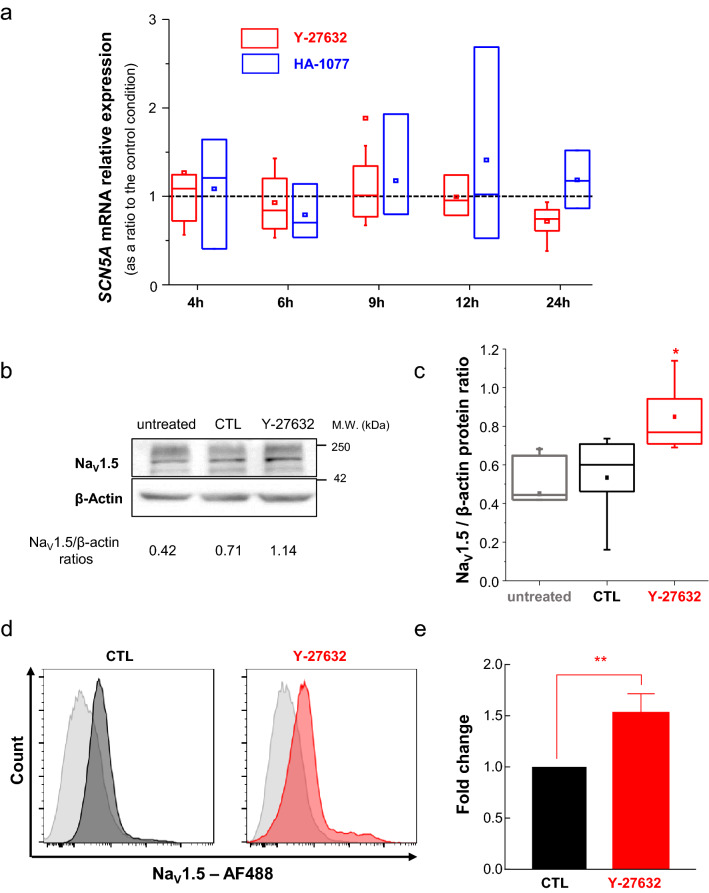
ROCK inhibitor Y-27632 increases Na_V_1.5 protein but not *SCN5A* gene expression. (**A**) mRNA expression levels of *SCN5A* gene assessed by RT-qPCR in SW620 colon cancer cells treated with Y-27632 (10 µM, red plots), or with Fasudil (20 µM, blue plots), at different times of treatment (ranging from 4 to 24 h), expressed as ratios to control conditions (vehicle, 0.1% DMSO) performed at the same time. There was no statistical difference, at any time, compared to the control condition represented as a dashed line. (**B**) Representative Western blotting analysis of Na_V_1.5 protein expression in untreated SW620 cells, or cells treated with vehicle (0.1% DMSO, CTL) or with 10 µM Y-27632 for 48 h. β-actin was used as loading control protein. This blot is representative of five independent experiments. (**C**) Change in Na_V_1.5 protein levels were studied by densitometric analyses of Western blotting experiments. Results are given as the ratio of Na_V_1.5 protein relative to β-actin for each condition. *P < 0.05 (Mann–Whitney rank sum test) compared to both untreated and CTL groups. (**D**) Representative histograms for SW620 cell population incubated with AF488 secondary antibody alone (gray histograms) and positive for Na_V_1.5 staining in absence (black histogram, CTL) or presence of the ROCK inhibitor (red histogram, Y-27632). (**E**) Mean fluorescence intensity (MFI) values obtained for the Y-27632 condition were averaged and relativized to the control condition. Plot shows fold-change calculated from three independent experiments *P < 0.05 (t-Student test).

To further explore the possible regulation of *SCN5A* expression by the ROCK signalling pathway, we measured its transcription level, by RT-qPCR, over a time range from 4 to 24 h treatment, with either Y-27632 or Fasudil treatments. Results obtained indicated no significant regulation of *SCN5A* expression by ROCK inhibitors at the mRNA level, during this time-scale (Fig. 3A). However, an increased level of Na_V_1.5 proteins was observed after 48 h treatment with Y-27632 (Fig. 3B–E). This appeared to be statistically increased by a median factor of 1.28, as compared to the CTL (vehicle) condition when assessed by western blotting experiments (Fig. 3C), and a significant increase in the mean fluorescence intensity (MFI) value by 1.52 times was recorded found under Y-27632 treatment by flow cytometry in non-permeabilized cells (Fig. 3E). This increased level of Na_V_1.5 proteins was also observed after 48 h treatment with Fasidul (Suppl. Figure 3A,B).

**Figure 4:**
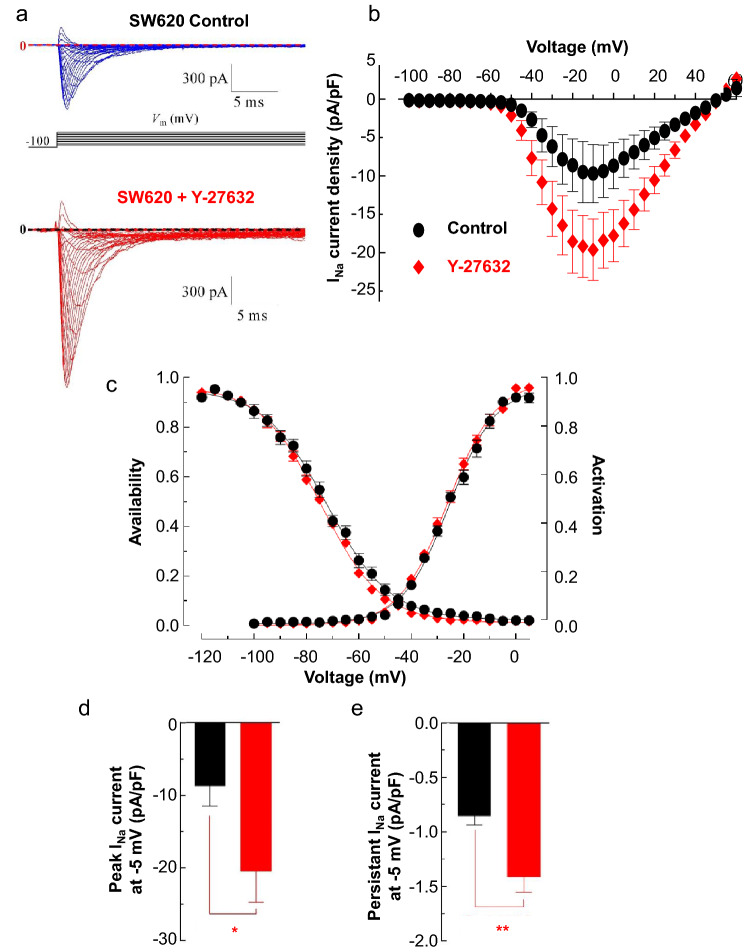
ROCK inhibitor Y-27632 increases Na_V_1.5 activity in SW620 colon cancer cells. (**A**) Representative whole-cell current recordings obtained from SW620 cells incubated with either vehicle (0.1% DMSO) as a Control (top recordings, blue traces), or 10 µM Y-27632 (bottom, red traces) for 48 h, in response to depolarizing 30-ms pulses from − 95 to + 60 mV in 5-mV steps applied every 2 s from a holding potential of − 100 mV. Dotted lines indicate baseline levels (zero current). (**B**) Sodium current density–voltage (I_Na_-V) relationship for Na_V_1.5 channels in SW620 cells in control (0.1% DMSO, n = 23) or 10 µM Y-27632 (n = 33). Peak sodium currents were averaged and plotted as a function of the membrane voltage. (**C**) Activation- and steady-state inactivation-voltage relationships of Na_V_1.5 currents recorded from SW620 cells in absence (control, black-filled circles) and presence (red-filled diamonds) of 10 µM Y-27632. Smooth lines correspond to the Boltzmann’s function fits and the V_1/2_ values obtained were the following: V_1/2_-activation voltage of − 23.4 ± 0.5 mV and − 26.1 ± 0.4 mV for control and Y-27632, respectively; and V_1/2_-inactivation of − 72.2 ± 0.6 mV and − 74.5 ± 0.4 mV for control and Y-27632, respectively. (**D**, **E**) Peak- and persistent sodium current densities evoked from a depolarizing step from -100 to − 5 mV in absence (black bars) and presence of 10 µM Y-27632 (red bars) obtained from the same cells than in B. *P < 0.05 and **P < 0.01 (Mann Whitney rank sum test).

This increased protein level at the cell surface, yet difficult to observe in immunocytochemistry experiments (Suppl. Figure 3C), was responsible for an increased Na_V_ activity at the plasma membrane of SW620 cells. Indeed, a 48 h-long treatment with Y-27632 was responsible for an increased amplitude of Na_V_-mediated transient inward currents (I_Na_) (Fig. 4A). There was no shift in the I_Na_-voltage relationship with a threshold of activation between − 60 and − 55 mV and a reversal potential between + 55 and + 60 mV, but a significant increase (*P* < 0.05) of the maximal amplitude (obtained for a depolarizing step from − 100 to − 10 mV), from − 9.68 ± 3.78 (n = 23) to − 19.61 ± 4.00 (n = 33) pA/pF in CTL and Y-27632 conditions, respectively (Fig. 4B). There was no significant change in both activation-voltage and inactivation-voltage relationships (Fig. 4C). There was a significant increase (*P* < 0.05) in the peak I_Na_ current density recorded from a holding potential of -100 to a depolarizing step of − 5 mV, from − 8.73 ± 2.75 (n = 22) to − 20.44 ± 4.29 (n = 33) pA/pF in Y-27632-treated cells (Fig. 4D). There was also a significant increase (*P* < 0.01) in the persistent I_Na_ current density recorded at the end of this same 30 ms-long depolarizing step, from − 0.86 ± 0.08 (n = 22) to − 1.42 ± 0.14 (n = 33) pA/pF (Fig. 4E), for CTL and Y-27632 conditions, respectively. These results therefore suggested that the inhibition of ROCK promotes Na_V_1.5 activity, by increasing the stability of the channel at the plasma membrane of cancer cells.

## Discussion

Voltage-gated sodium channels (Na_V_) are membrane spanning heteromeric complexes, composed of one large pore-forming α subunit (9 isoforms, Na_V_1.1–1.9) associated with one or two smaller auxiliary β subunits (4 transmembrane β1-4, and one soluble β1B, isoforms), traditionally considered as features of excitable cells, because of their well-characterized participation in the generation of action potentials. However, it has been recognized that these channels are also expressed, and fully functional, in several carcinoma cells^30,44–50^, where they are not associated with cellular excitability but rather to dedifferentiation of epithelial cells^43^, invasive properties and metastatic potencies^37,38,51^. Several Na_V_ isoforms, mostly Na_V_1.5, Na_V_1.6 and Na_V_1.7 depending on the cancer type^52^, have been shown to be abnormally expressed, but the origin of this dysregulated expression as well as the reasons for the association with a specific cancer tissue have not been identified. Whether these channels are regulated by intracellular signalling pathways in cancer cells is still unclear, and has not been fully characterized.

The Na_V_1.5 isoform, which is the product of the *SCN5A* gene, was found to be highly overexpressed at both mRNA and protein levels in colon and breast tumours, compared to normal tissues, and was correlated with cancer recurrence, metastases development and reduced patients survival^30,42,53,54^. In tumours, Na_V_1.5 was expressed and functional at the plasma membrane of cancer cells, thus giving rise to sodium currents, but the isoform expressed was a neonatal splice variants^32,54^, and not the adult splice variant isoform. This splice variant only differs from the adult one by a few amino acid residues. In colon and breast cancers, the activity of Na_V_1.5 results in a small but persistent entry of Na^+^ at the basal membrane potential, that was demonstrated to promote extracellular matrix degradation, cancer cell invasiveness in vitro^30,31,36,39,44,55^, primary tumour growth and metastases development in animal models^40,41^. This paved the way for the development of new small inhibitors of Na_V_^56^ or to the repurposing of clinically used inhibitors for anticancer treatments^32,40,41,57,58^. On the opposite, drugs promoting Na_V_ activity in cancer cells, such as the alkaloid veratridine, importantly increase invasive behaviours in in vitro experiments^31,32,44^.

In this study, we show that the inhibition of ROCK, using conventional ROCK inhibitors at classical concentrations used both in vitro and in vivo, increases the invasive capacities of SW620 human colon cancer cells, and also those of MDA-MB-231 human breast cancer cells. While ROCK inhibitors are generally used to inhibit cell migration and invasion^7,8,19^, we are not the first to demonstrate pro-invasive effects of ROCK inhibition^25,29^. In B16 melanoma cells, Y-27632 induced invasion via enhanced AKT and ERK signalling pathways^25^. In SW620 colon cancer cells, Y-27632 was also shown to increase invasiveness through 3D matrices composed of collagen I, but the mechanisms involved were not identified^29^.

In this study, we show that the two well-known and commonly used pharmacological inhibitors of ROCK, Y-27632 and Fasudil, both promoted 2D and 3D-invasive capacities, through both Matrigel- or collagen-I-composed extracellular matrices. Importantly, an increased invasion was also confirmed when silencing either ROCK-1 or ROCK-2, using specific siRNA, thus ruling out non-specific effects of the pharmacological compounds. Pro-invasive effects of ROCK inhibition were importantly abrogated by the concomitant inhibition of Na_V_1.5 channel, identifying this channel as a potential target of the ROCK signalling. Indeed, the activity of Na_V_1.5 channel, i.e. peak and persistent sodium currents, was demonstrated to be increased with no change in voltage-dependencies for the activation or for the inactivation. This suggests an increased quantity of Na_V_1.5 proteins at the plasma membrane. Interestingly, the inhibition of ROCK did not interfere with *SCN5A* gene expression, and there was no effect at the mRNA level. However, an increased level of Na_V_1.5 proteins under ROCK inhibition treatment could be identified, thus favouring the hypothesis of an increased stability and half-life of Na_V_1.5 proteins, maybe by reducing their recycling.

While the inhibition of ROCK increased Na_V_1.5 activity and related invasive capacity in SW620 colon cancer cells, it could appear surprising that the inhibition of RhoA expression induced no effect on cell invasion. Indeed, ROCK-1 and ROCK-2 are well-known effectors of activated RhoA, but could also be activated by RhoC^59^. Our results may also appear to be in contradiction with results obtained in MDA-MB-231 breast cancer cells, in which RhoA silencing reduced cell invasiveness by reducing the mRNA expression of *SCN5A* and therefore Na_V_1.5-mediated sodium current, thus indicating that RhoA could exerts a tonic effect on the expression of Nav1.5 in these cancer cells^60^. Furthermore, in the same study, the authors identified the existence of a positive feedback of Nav1.5 channel expression on that of RhoA^60^. In our study, performed in a different cell type, we did not measure the effect of RhoA silencing on Na_V_1.5 currents, but identified no significant effect on cell invasiveness. This could possibly be explained by a different stoichiometry in Rho GTPase or by different levels of basal activation.

Our results also appear in apparent contradiction with those obtained by Vishnubhotla and collaborators who reported that ROCK-2 was highly expressed in SW620 colon cancer cells, mainly distributed in invadopodial-like structures and that its knock-down reduced the depth of invasion into a 3D-scaffold of type I collagen^61^. However, our results are in line with those recently reported by Libanje and collaborators who demonstrated that colorectal cancer cells mainly harbour a collective mode of invasion and that ROCK-2 inhibition triggers the initial induction of leader cell formation and induces collective invasion from cysts^62^. Therefore, these apparently contrasting results should probably be studied with regard to the different growing modes and environmental conditions (2D vs 3D, type and stiffness of the extracellular matrix, etc.) and the different modes of invasion that could be induced in these situations.

Taken together, our results clearly demonstrated that the inhibition of ROCK could induce procancerous effects, and most specifically promote cancer cell invasion. These results also identify Na_V_1.5 channels as potential targets of ROCK. Therefore, the use of ROCK inhibitors for anticancer purposes should be tightly controlled and probably restricted to cancers in which Na_V_ channels have not been identified, in order to avoid adverse effects.

## Methods

### Chemicals, antibodies and pharmacological compounds

Tetrodotoxin was purchased from LATOXAN (France) and used at the final concentration of 30 µM prepared in PBS, thus blocking the activity of Na_V_1.5 channels^44^. Y-27632 was purchased from SELLECKCHEM (France) and used at the final concentration of 10 µM (prepared in 0.1% DMSO), a conventional concentration used both in vitro and in vivo experiments^33,34^. Fasudil (HA-1077) was purchased from SIGMA-ALDRICH (France) and used at the final concentration of 20 µM prepared in a saline solution (PBS) as previously reported^63^. Fluorescent probes and conjugated antibodies were purchased from THERMOFISHER SCIENTIFIC (France). All other drugs and chemicals were purchased from SIGMA-ALDRICH (France).

### Cell lines and culture

SW620 human colon and MDA-MB-231 human breast cancer cell lines were purchased from the American Type Culture Collection (LGC PROMOCHEM, France). Cancer cells were grown at 37 °C in a 5% CO_2_ incubator, in a humidified atmosphere. MDA-MB-231 breast cancer cells were cultured in DMEM supplemented with 5% foetal calf serum (FCS). SW620 colon cancer cells were cultured in DMEM supplemented with 10% FCS. SW620 shCTL and shNa_V_1.5 stable colon cancer cells, respectively expressing a null-target and a *SCN5A* expression product-targeting small hairpin RNA were generated as previously described^40,64^ using the Giga Viral vectors Plateform (University of Liège, Belgium). Briefly, these two cell lines were obtained by transduction with a lentiviral vector encoding a short hairpin RNA (shRNA) specifically targeting human *SCN5A* transcripts (shNa_V_1.5 cell line) or a null-target shRNA (shCTL cell line). The sequence encoding sh*SCN5A*, inhibiting the expression of Na_V_1.5 protein, was 5′-GCTGGACTTTAGTGTGATTATCTCGAGATAATCACACTAAAGTCCAGC-3′. We constructed a lentiviral vector expressing a null-target shRNA (pLenti-shCTL), with the following sequence: 5′-CCTAAGGTTAAGTCGCCCTCGCTCGAGCGAGGGCGACTTAACCTTAGG-3′.

Tests assessing for mycoplasma contamination were performed once a week (LONZA, MycoAlert Mycoplasma Detection Kit).

### RNA extraction, Reverse transcription, and real-time PCR

Total RNA extraction was performed (RNAgents Total RNA Isolation System, PROMEGA, France) and RNA yield and purity were determined by spectrophotometry (NanoDrop 2000, THERMO SCIENTIFIC, France). Only samples with a A260/A280 ratio above 1.6 were kept for reverse-transcription. To do so, RT kits Ready-to-go You-prime First-Strand Beads (AMERSHAM BIOSCIENCES, UK) and random hexamers pd(N)_6_ 5′-Phosphate (0.2 µg, AMERSHAM BIOSCIENCES) were used. Samples were incubated at 37 °C for 60 min. Real time PCR experiments were performed as previously described^44^. Results obtained from cell lines are expressed as the relative gene expression using the comparative 2^-ΔΔCt^ method^65^ with *PPIA* and *HPRT1* as reference genes. Primers sequences can be found in Table 1.

**Table 1:** PCR primers sequences and expected amplicon size.

Gene	Protein	Forward primers (5′ → 3′)	Reverse primers (5′ → 3′)	Expected size (bp)
*HPRT1*	Hprt1	TTGCTGACCTGCTGGATTAC	TATGTCCCCTGTTGACTGGT	119
*PPIA*	Peptidylprolyl isomerase A (PPIA) Cyclophilin A	ACCGCCGAGGAAAACCGTGTA	TGCTGTCTTTGGGACCTTGTCTGC	129
*SCN5A*	Na_V_1.5	TTCCTGGGGTCCTTCTACCT	TTTCCTTCTCCTCGGTCTCA	103
*ROCK1*	ROCK1	AGCGGTTGGAACACCTGATT	AACCGACCACCAGTCACATT	94
*RHOA*	RhoA	CGCTTTTGGGTACATGGAGT	GAGCAGCTCTCGTAGCCATT	80

### Transfection of small interfering RNA

SW620 human breast colon cells were transfected with 20 nM small interfering RNA (siRNA, TEBU-BIO, France) targeting the expression of *ROCK1* (siROCK1, sc-29473, mix of three different sequences), *ROCK2* (siROCK2, sc-2947420, mix of three different sequences) or targeting the expression of *RHOA* (siRHOA, sc-29471, mix of three different sequences), or scrambled siRNA (siCTL, siRNA-A sc-37007). SiRNA Transfections were performed with Lipofectamine RNAi max (INVITROGEN, France) when cells reached 80–90% confluence. Transfection efficiency was verified by qPCR using an iCycler system (BIORAD, USA).

### Cellular electrophysiology

Whole-cell currents were recorded, as already described^55^, under the voltage-clamp mode of the patch-clamp technique, at room temperature, using an Axopatch 200B patch clamp amplifier (AXON INSTRUMENT, USA). Patch pipettes were pulled from borosilicate glass (TW150-3, WORLD PRECISION INSTRUMENTS, France) to a resistance of 3–5 MΩ. Analogue signals were filtered at 5 kHz, and sampled at 10 kHz using a 1440A Digidata converter. Cell capacitance and series resistance were electronically compensated by about 60%. The P/2 sub-pulse correction of cell leakage and capacitance was used to study Na^+^ current (I_Na_). Peak and persistent sodium currents were recorded by depolarizing the cells from a holding potential (HP) of − 100 mV to a test pulse of − 5 mV for 30 ms every 500 ms, and the amplitude of the persistent current was measured at the end on this 30 ms-long protocol. Sodium current–voltage (I_Na_-V) relationships were determined by stepwise depolarizing the membrane from a HP of − 100 mV to a maximal voltage of + 60 mV, with 5-mV increments, for 50 ms and at a frequency of 2 Hz. Inactivation-voltage relationships were obtained by applying 50 ms-long prepulses using the I_Na_–V curve procedure, followed by a depolarizing pulse to − 5 mV for 50 ms. Currents were normalized to the amplitude of the test current without a prepulse. Currents amplitudes were normalized to cell capacitance and expressed as current density (pA/pF). The bath solution had the following composition (in mM): NaCl 140, KCl 4, MgCl_2_ 1, CaCl_2_ 2, D-Glucose 11.1, and HEPES 10, adjusted to pH 7.4 with NaOH (1 M). The intrapipette solution had the following composition (in mM): KCl 130, NaCl 15, CaCl_2_ 0.37, MgCl_2_ 1, Mg-ATP 1, EGTA 1, HEPES 10, adjusted to pH 7.2 with KOH (1 M).

### Cell viability

SW620 cancer cells were seeded in a 24-well plate at the density of 15.10^3^ cells per well. Media were changed every day, and after 5 days growing, the number of viable cells was assessed by the tetrazolium salt assay as previously described^44^ and normalised to the appropriate control condition (vehicle, 0.1% DMSO). Acquisitions were taken at 570 nm using a spectrophotometer TECAN Nanoquant Infinite 200 Pro (France).

### Three-dimensions (3D) invasion model

3D-spheroids were generated from the SW620 human colon cancer cell line using 96-well round-bottom Ultra Low Attachment (ULA) plates (CORNING, New York, USA) inhibiting cell attachment. Briefly, 500 cells/ well were seeded in 200 µl DMEM + 10% FCS in ULA plates. Forty-eight hours later, when spheroid were formed and visible, Matrigel at a final concentration of 300 µg/mL was added to the culture medium. Four hours after the addition of Matrigel, treatments were performed, adding 50 μL of fresh culture medium with the indicated compounds (10 µM Y-27632 and/or 30 µM TTX) into each well. Plates were then centrifuged at 300×*g* for 3 min, and placed on the motorized stage of the NIKON TI-E microscope (NIKON, France) equipped with a time lapse system, in a controlled atmosphere chamber at 37 °C, 5% CO_2_ and saturated with humidity. Images were taken every 30 min, for a total period of 96 h of acquisition at a 100× magnification, using the NIKON DS-Qi2 camera connected to the microscope. Spheroid growth and morphology were analysed using Fiji software (https://imagej.net/Fiji, National Institute of Health, USA). Several parameters were evaluated after having manually delineated the shape of spheroids: circularity index, spherical volume and matrix invasion area. Spherical volume (V) was estimated by measuring spheroid perimeter (P) and calculated with the formula V = P^3^/6π^2^. Matrix invasion area was evaluated by subtracting the invasion area at time t from the initial spheroid area at t = 0.

### Two-dimensions cancer cell invasiveness

Cancer cell invasiveness was assessed using culture inserts with 8-µm pore size migration filters (BECTON DICKINSON, France), that we covered with a film of Matrigel (500 µg/mL) or collagen I (300 µg/mL). The upper chamber of the insert was then seeded with 1 × 10^5^ cells in 200 µL DMEM supplemented with 0.1% FCS, and the lower compartment was filled with 800 µL DMEM supplemented with 10% FCS, thus creating a chemoattractant gradient. Cells invasion was allowed for 48 h at the 37 °C and 5%-CO_2_ incubator. Cells that had invaded and had reached the underneath surface of the filter were stained with DAPI, then counted on the whole area of the insert membrane using the Binary/Bright spot tool of NIS-Elements V4.30.02 software (NIKON, France). Assays were performed in triplicate in each separate experiment.

### Western blotting experiments

Cells were washed twice with PBS and lysed in presence of a lysis buffer (50 mM Tris, pH 7, 100 mM NaCl, 5 mM MgCl_2_, 10% glycerol, 1 mM EDTA), containing 1% Triton-X-100 and protease inhibitors (SIGMA-ALDRICH, France). Cell lysates were cleared by centrifugation at 16,000×*g* for 10 min. Total protein concentrations were determined using the Pierce® BCA Protein Assay Kit (THERMO FISHER SCIENTIFIC, France). Protein sample buffer was added and the samples were boiled at 100 °C for 3 min. Total protein samples were electrophoretically separated by sodium dodecyl sulphate–polyacrylamide gel electrophoresis in 10% gels, and then transferred to polyvinylidene fluoride membranes (MILLIPORE, USA). Na_V_1.5 proteins were detected using anti-human Na_V_1.5 rabbit polyclonal primary antibodies (1/1000, Ref S0818, SIGMA-ALDRICH) and horseradish peroxidase (HRP)-conjugated goat anti-rabbit IgG secondary antibody at 1:2000 (TEBU-BIO, France). ROCK-1 and ROCK-2 proteins were detected using HRP-conjugated primary antibodies (SANTA CRUZ references G-6 sc-17794 and D-11 sc-398519, respectively) used at the working dilution of 1/1000. HSC70 protein was detected as a sample loading control using anti-HSC70 mouse primary antibody at 1:30,000 (TEBU-BIO) and HRP-conjugated anti-mouse-IgG secondary antibodies at 1:2000 (TEBU-BIO). In some other conditions β-actin was used as a sample loading control using anti-β-actin-HRP primary antibody at 1:1000 (C4, SANTA CRUZ ref sc-47778).

Proteins were revealed using electrochemiluminescence-plus kit (PIERCE ECL Western Blotting Substrate, THERMO FISHER SCIENTIFIC, France) and captured on a PXi acquisitions system (SYNGENE, UK). Densitometry analysis of protein bands was performed using the Gel Tool from Fiji software (Scientific image analysis software available at https://fiji.sc). All original blots are provided in Supplementary Figs. 4–7.

### Epifluorescence imaging

SW620 colon cancer cells were cultured for 24–48 h on glass coverslips, before receiving ROCK inhibitor (or vehicle treatment) for a duration of 48 h. Cells were then washed twice in PBS, before being fixed with 3.7% ice-cold paraformaldehyde prepared in PBS. Cell permeabilization was obtained using a solution containing 50 mM NH_4_Cl, 1% BSA and 0.02% saponin. Saturation of epitopes was achieved by incubating for 2 h with a solution containing 3% BSA and 3% Normal Goat Serum (NGS). Epifluorescence microscopy was performed with a NIKON TI-S microscope, and images were analysed using the NIS-BR software (NIKON, France). Fluorescent probes and conjugated antibodies were purchased from THERMO FISHER SCIENTIFIC (France).

### Flow cytometry

SW620 cancer cells were seeded in T-25 flasks at the density of 1 × 10^6^ cells per flask. Twenty-four hours after cell seeding, treatments were performed, adding 0.1% DMSO (vehicle, control) or 10 µM Y-27632 into each flask. Cells were incubated for 48 h and detached using accutase solution. Cells were re-suspended in PBS supplemented with EDTA and 0.1% FBS. For each condition, 4 × 10^5^ cells were incubated with 1.6-μg anti-Na_V_1.5 antibody (ASC-005, ALOMONE LABS, Israel) for 30 min at 4 °C followed by incubation with secondary antibody Alexa-Fluor-488 (1:500, A-11008, THERMO FISHER SCIENTIFIC, France) for 45 min at 4 °C. Background controls were obtained after incubation with secondary antibody alone. All data were performed using a BD FACSMelody cell sorter (BECTON DICKINSON, San Jose, USA) and analyzed using FlowJo software (TREE STAR, USA).

### Statistical analyses

Statistical analyses were performed using SigmaStat 3.0 software (SYSTAT SOFTWARE INC.). Normality of sample distribution was tested prior to conduct any comparison between groups. When normality failed, and/or equal variance test failed, non-parametric statistical tests were performed (Mann–Whitney rank sum test) and data were displayed as box plots indicating the first quartile, the median, and the fourth quartile, whiskers indicating the minimal and maximal values, and square dots indicating the means. When normality and equal variance were obtained, parametric tests (Student’s t test) were performed. In these cases, results were presented as mean ± SEM. *P* values are indicated on figures. NS stands for "not statistically different".

## Supplementary information

Supplementary Information.

## Data Availability

Requests for data our materials should be addressed to S.R. (sebastien.roger@univ-tours.fr).
