# Supplementary material for: Rock inhibition promotes NaV1.5 sodium channel-dependent SW620 colon cancer cell invasiveness
Source: Sci Rep. 2020 Aug 7;10:13350. doi: 10.1038/s41598-020-70378-3 (PMC7414216; doi:10.1038/s41598-020-70378-3)

SUPPLEMENTARY FIGURE LEGENDS

ROCK INHIBITION PROMOTES *NaV1.5* SODIUM CHANNEL-DEPENDENT SW620 COLON CANCER CELL INVASIVENESS

Lucile POISSON[1,2*], Osbaldo LOPEZ-CHARCAS[1*], Stéphanie CHADET[1], Emeline BON[1], Roxane LEMOINE[1], Lucie BRISSON[2], Mehdi OUAISSI[1,3], Christophe BARON[1,3], Pierre BESSON[1§], Sébastien ROGER[1,4§] & Driffa MOUSSATA[1,3§]

[1] EA4245 Transplantation, Immunologie, Inflammation ; Université de Tours, France

[2] Inserm UMR1069, Nutrition, Croissance et Cancer ; Université de Tours, France

[3] CHRU de Tours, France

[4] Institut Universitaire de France, Paris, France

Supplementary Figure 1: Effect of tetrodotoxin (TTX) on the inhibition of Na_V currents in SW620 colon cancer cells. The figure is showing representative traces of fast inward sodium currents for a membrane depolarizing step from -100 to -5 mV for a duration of 50 ms (only the first 5 ms are shown here) in a SW620 cancer cell, in normal physiological saline solution (PSS, black trace) or in presence of 300 nM, 3 µM or 30 µM tetrodotoxin (TTX, red traces), or when extracellular Na+ was substituted by N-Methyl D-Glucamin (0-Na^+, blue trace).

Supplementary Figure 2: Effect of ROCK inhibitors on cancer cell viability and invasiveness.

A, SW620 colon cancer cell viability was measured after 96 hours incubation in presence of Y-27632 (10 µM), TTX (30 µM) or a combination of both (Y-27632 +TTX) and expressed relatively to the control condition (CTL, vehicle). Results are coming from 3 independent experiments, and are expressed as mean ± standard error to the mean (SEM). There was no statistical difference. **B**, SW620 colon cancer cell viability was measured after 96 hours incubation in presence of Fasudil (20 µM), TTX (30 µM) or a combination of both (Fasudil +TTX) and expressed relatively to the control condition (CTL, vehicle). Results are from 3 independent experiments, and are expressed as mean ± standard error to the mean (SEM). Fasudil alone statistically reduced cell viability compared to the control condition at 48h, 72h and 96h (*, $P<0.05$, Mann-Whitney Rank sum test). Similarly, the combination of Fasudil + TTX statistically reduced cell viability compared to the control condition at 48h, 72h and 96h (*, $P<0.05$, Mann-Whitney Rank sum test). There was no statistical difference between Fasudil and Fasudil + TTX treatments**. C**, Representative micrographs of MDA-MB-231 human breast cancer cell in control condition (vehicle 0.1% DMSO, CTL) or treated for 24h, 48h or 72h in presence of 30 µM TTX, 10 µM Y-27632 or 20 µM Fasudil. Scale bar, 30 µm. Right, a cell circularity index was calculated from micrographs at the three different times (n=100 cells for each condition). This was performed using the Fiji software after having manually delineated the shape of cells. *** indicates a statistical difference at $P<0.001$ (Mann-Whitney rank sum test) versus CTL condition of corresponding time. NS stands for not statistically different. **D**, MDA-MB-231 human breast cancer cell invasiveness through Matrigel-coated inserts in absence (CTL, vehicle) or presence of Y-27632 (10 µM). Results are from 10 independent experiments. ***, $P<0.001$ (Mann-Whitney Rank sum test).

Supplementary Figure 3: Effect of ROCK inhibitor treatments on $Na_V1.5$ protein expression in SW620 human cancer cells.

A, Representative western blotting analysis of $Na_V1.5$ protein expression in untreated SW620 cells, or cells treated with vehicle (0.1% DMSO, CTL) or with 20 µM Fasudil for 48 h. HSC70 was used as loading control protein. This blot is representative of four independent experiments. **B**, Change in $Na_V1.5$ protein levels were studied by densitometric analyses of Western blotting experiments. Results are given as the ratio of $Na_V1.5$ protein relative to HSC70 for each condition (n= 6 independent experiments). *, $P < 0.05$ (Mann-Whitney rank sum test) compared to CTL group, and **, $P < 0.01$ compared to untreated group. **C**, Immunofluorescence microscopy analysis of $Na_V1.5$ protein expression in SW620 cells in absence (CTL, vehicle) or presence of Y-27632 (10 µM). SW620 cells were stained for $Na_V1.5$, using specific NaV1.5 primary antibodies, followed by membrane labeling with WGA (Wheat Germ Agglutinin). DAPI reagent was used for nucleus staining. Imaging acquisition was performed with an inverted epifluorescence microscope. Scale bar, 50 µm.

Supplementary Figure 4: Original uncropped western blots presented in Figure 2D

A, Full, uncropped western blots used for the left part of figure 2D, showing ROCK-1 and β-actin bands in the different treatment conditions indicated above the bands. The square in the blots represent the portion of the blots shown in figure 2D, left. **B**, Full, uncropped western blots used for the right part of figure 2D, showing ROCK-2 and β-actin bands in the different treatment conditions indicated above the bands. The square in the blots represent the portion of the blots shown in figure 2D, right.

Supplementary Figure 5: Original uncropped western blots presented in Figure 2F

Full, uncropped western blots used for the left part of figure 2F, showing $Na_V1.5$ and β-actin bands in shCTL and shNaV1.5 SW620 cell lines and in rat heart protein extract (used as a positive control). The square in the blots represent the portion of the blots shown in figure 2F, right.

Supplementary Figure 6: Original uncropped western blots used in Figure 3

A, Full, uncropped western blots used for figure 3B, showing $Na_V1.5$ and β-actin bands in the different treatment conditions indicated above the bands. The red square in the blots represent the portion of the blot shown in figure 3B. **B**, All other blots which have been used for the

densitometry quantification of $Na_V1.5$ proteins in the different treatment conditions. Green squares are region of interest which have been studied for densitometry.

Supplementary Figure 7: Original uncropped western blots used in Suppl. Figure 3. Representative western blotting analysis of $Na_V1.5$ protein expression in untreated SW620 cells, or cells treated with vehicle (0.1% DMSO, CTL) or with 20 µM Fasudil for 48 h. HSC70 was used as loading control protein. This blot is representative of four independent experiments.

Suppl. Figure 1

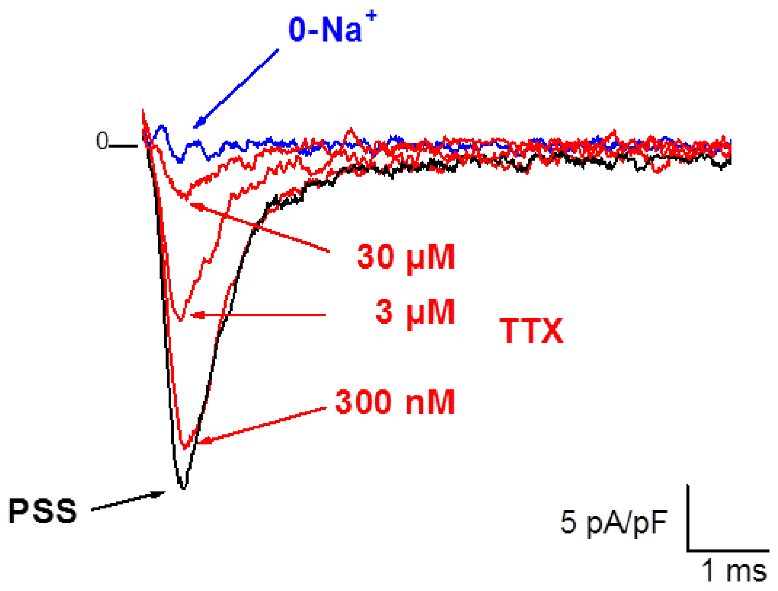

Suppl. Figure 2

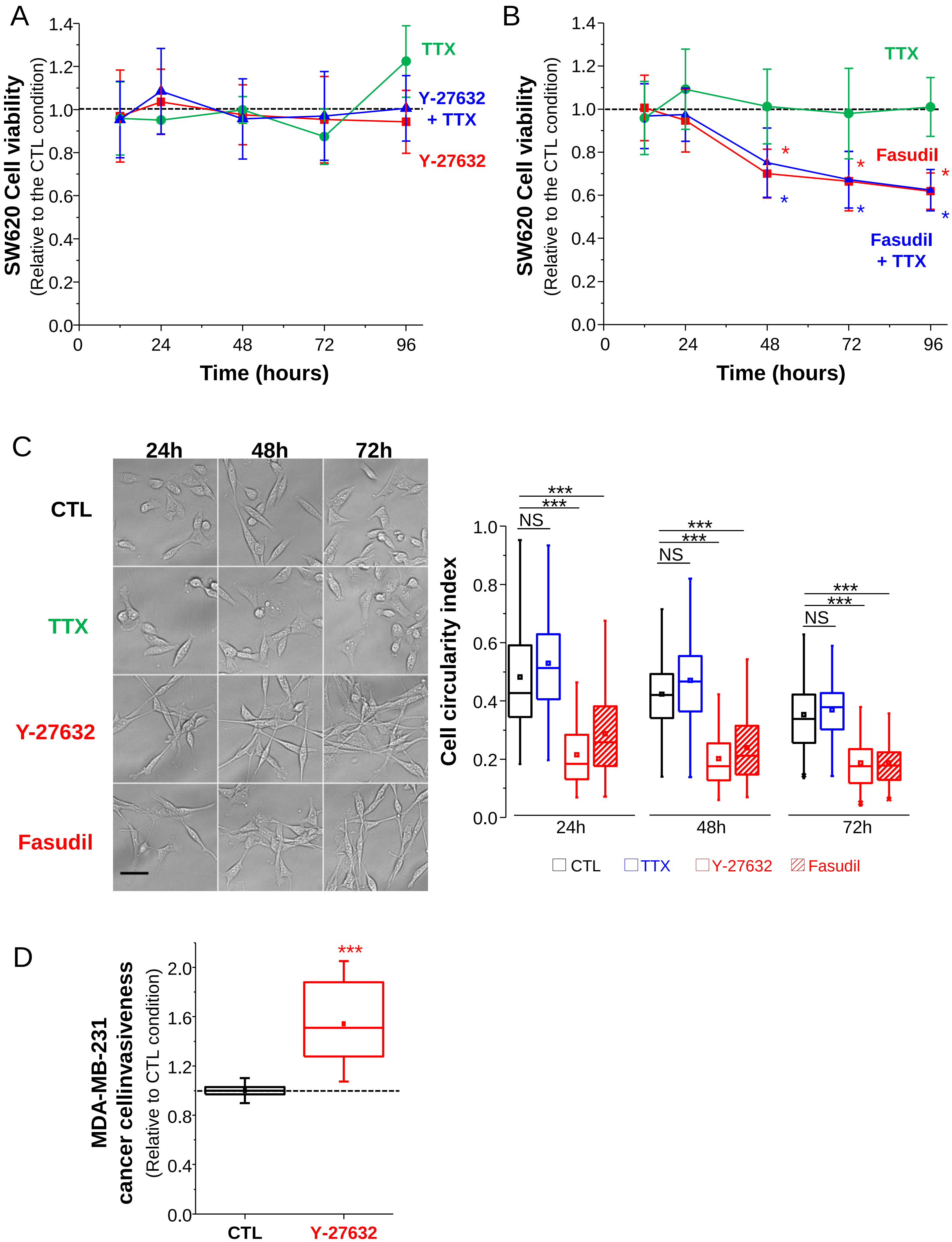

Suppl. Figure 3

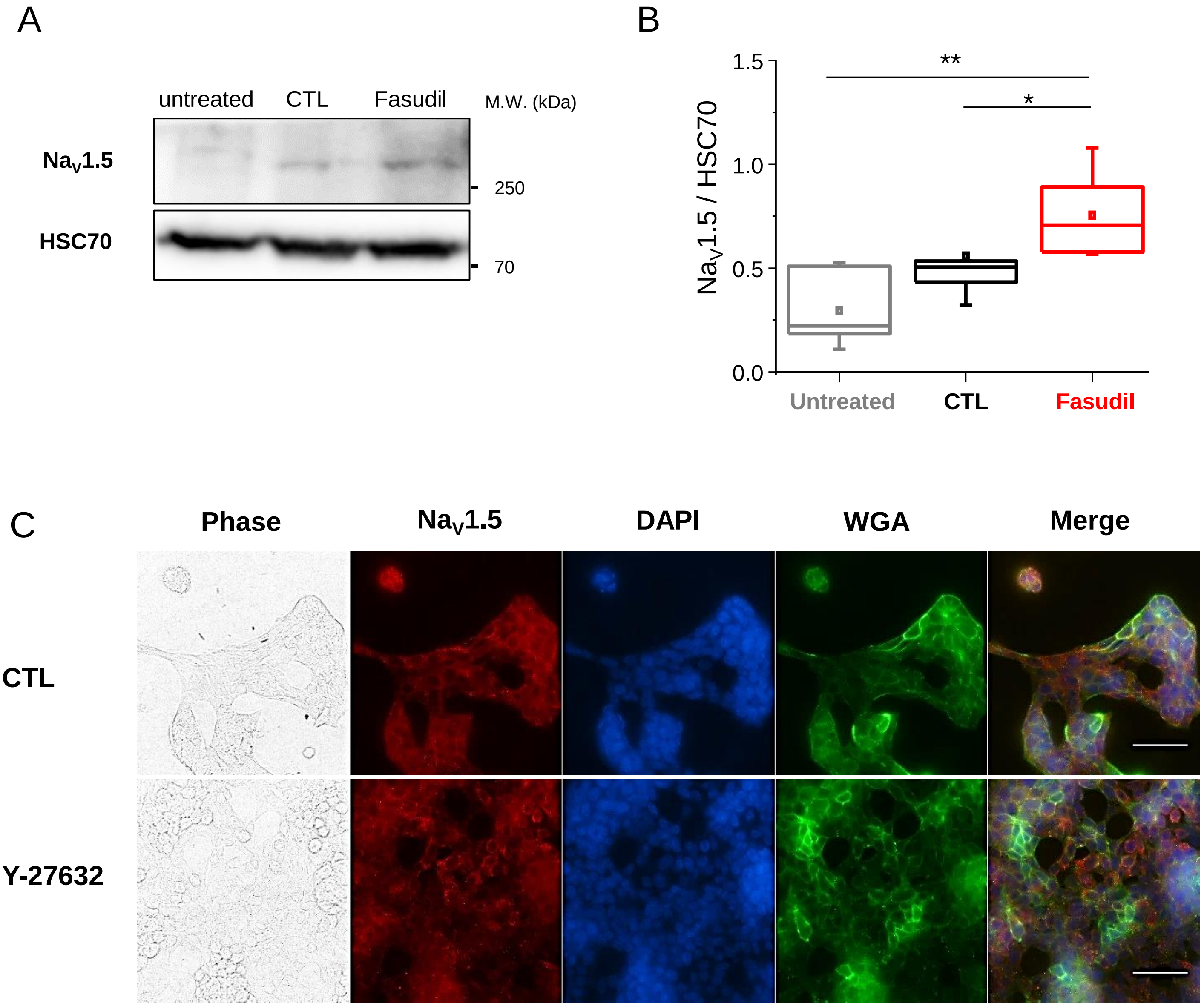

Suppl. Figure 4 – Blots shown in Figure 2D

A

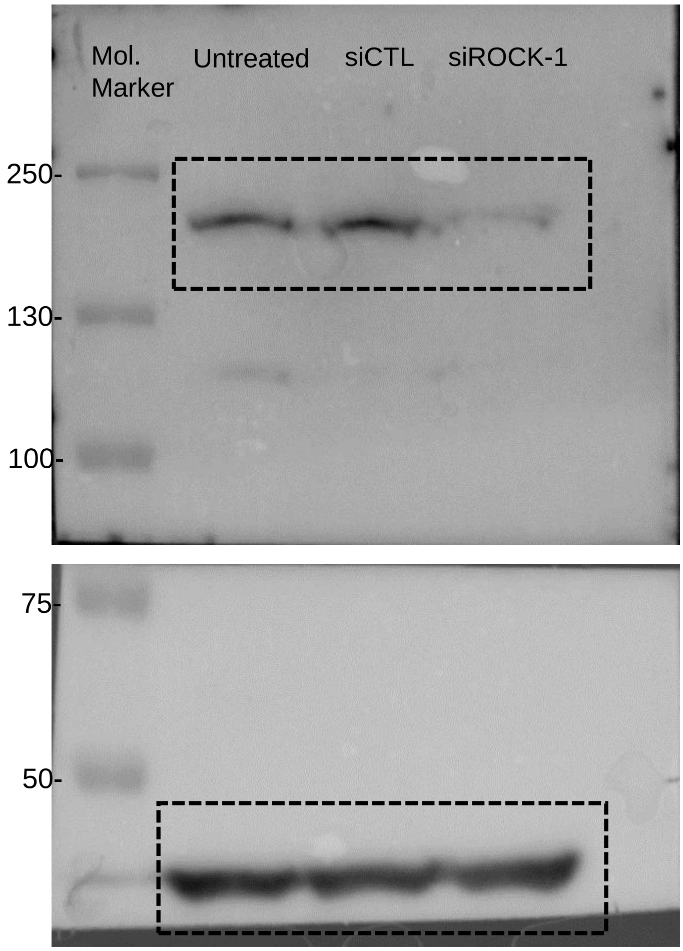

B

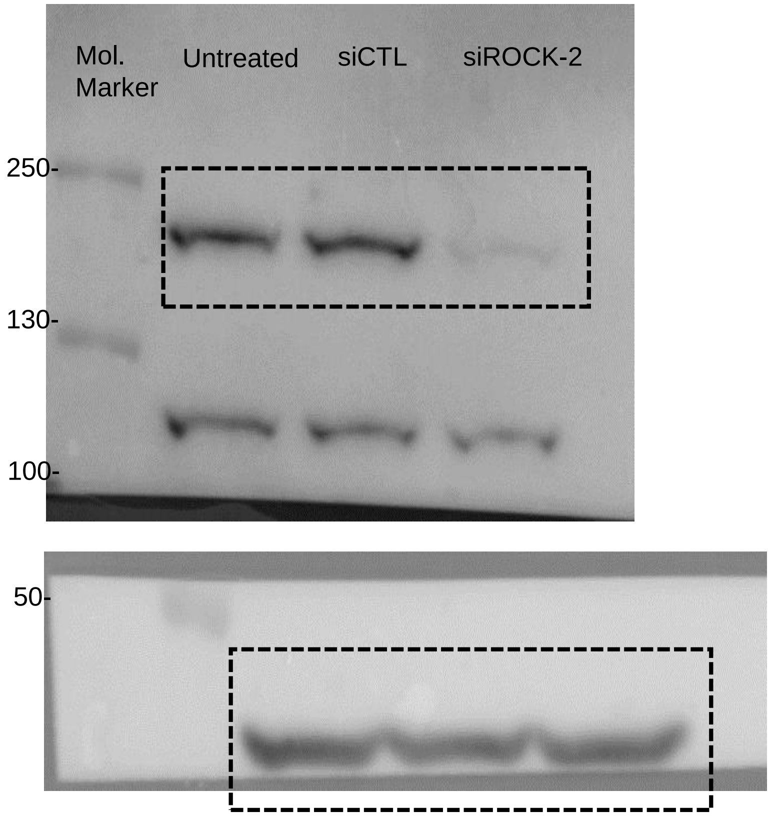

Suppl. Figure 5 – Blots shown in figure 2F

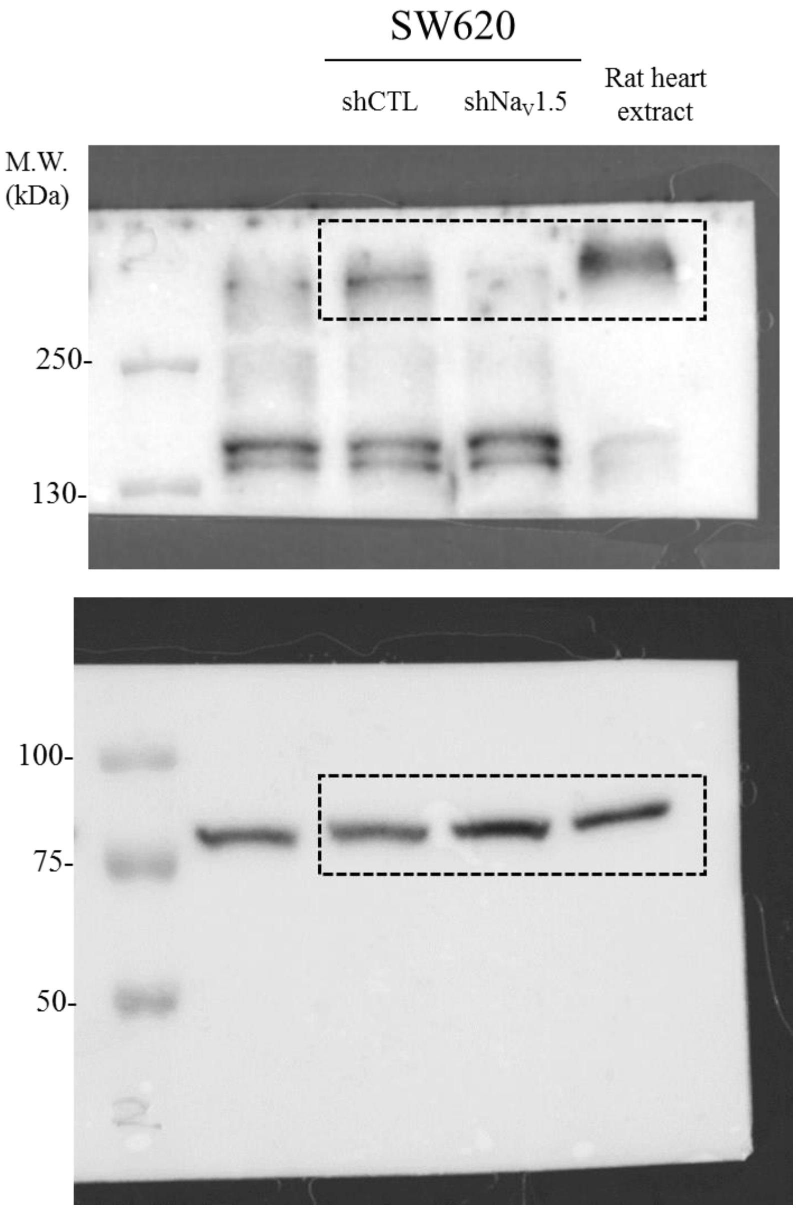

Suppl. Figure 6 – Blots shown in figure 3B

A

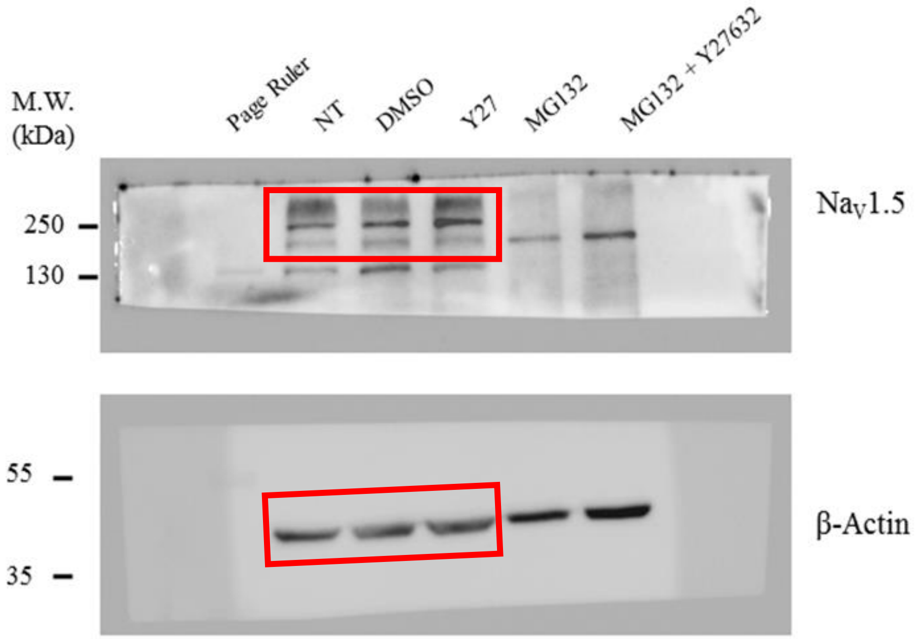

B

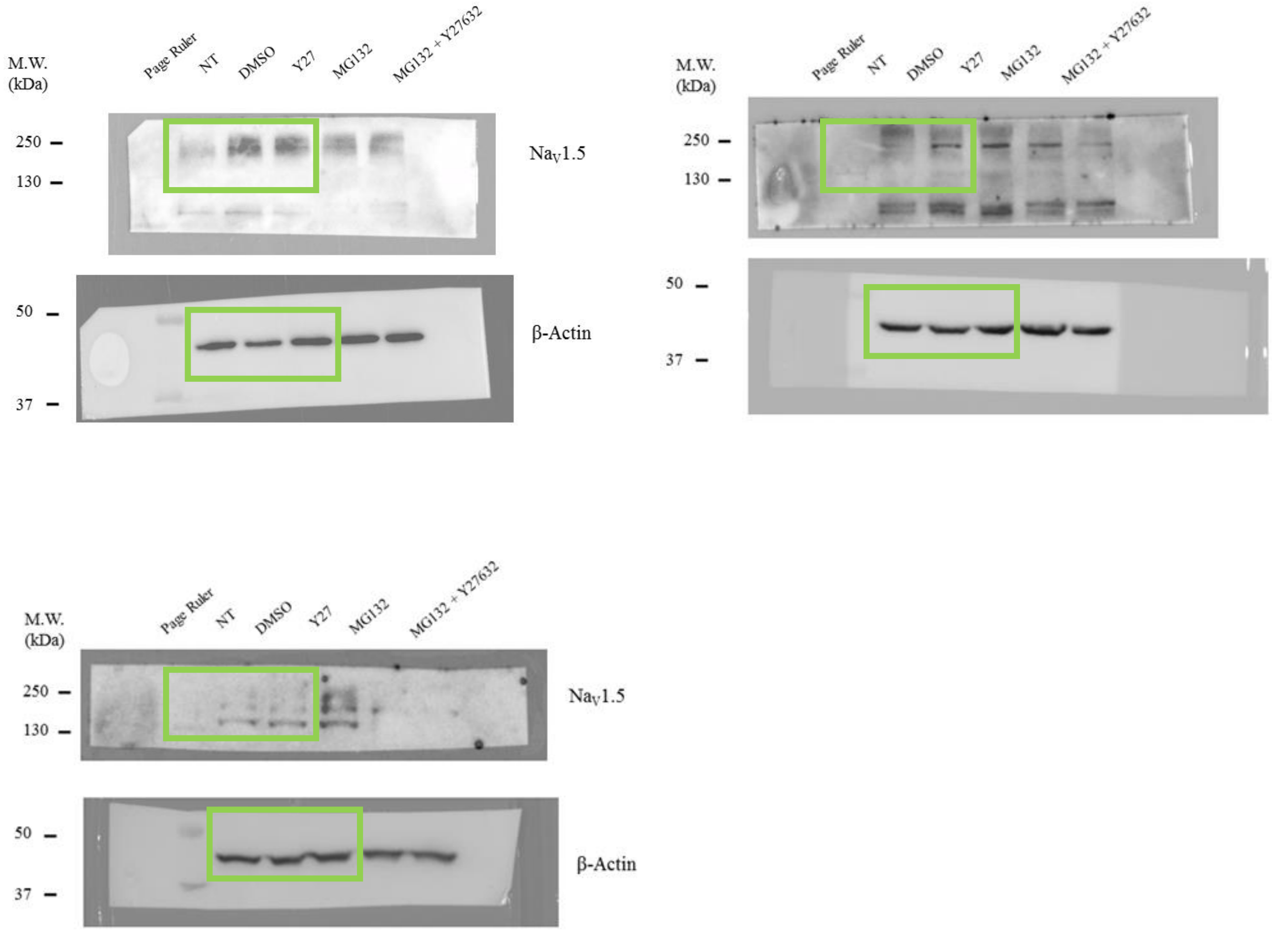

Suppl. Figure 7 – Blots shown in Suppl figure 3B

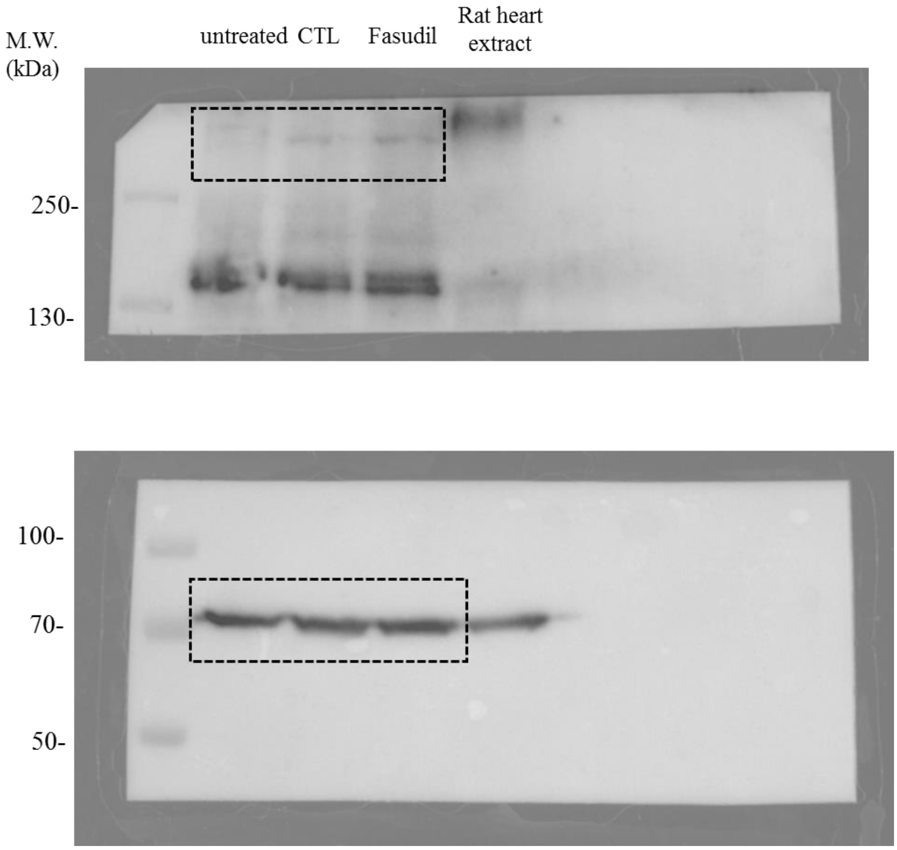

Supplement: Supplementary file 1 — Supplementary Information. [file 41598_2020_70378_MOESM1_ESM.pdf]
